# Genome-wide analysis of population structure, effective population size and inbreeding in Iranian and exotic horses

**DOI:** 10.1371/journal.pone.0299109

**Published:** 2024-03-05

**Authors:** B. Bazvand, A. Rashidi, M. B. Zandi, M. H. Moradi, J. Rostamzadeh

**Affiliations:** 1 Department of Animal Science, Faculty of Agriculture, University of Kurdishistan, Sanandaj, Kurdishistan, Iran; 2 Department of Animal Science, Faculty of Agriculture, University of Zanjan, Zanjan, Iran; 3 Department of Animal Science, Faculty of Agriculture and Natural Resources, Arak University, Arak, Iran; Long Island University - CW Post Campus: Long Island University, UNITED STATES

## Abstract

Population structure and genetic diversity are the key parameters to study the breeding history of animals. This research aimed to provide a characterization of the population structure and to compare the effective population size (N_e_), LD decay, genetic diversity, and genomic inbreeding in Iranian native Caspian (*n* = 38), Turkmen (*n* = 24) and Kurdish (*n* = 29) breeds and some other exotic horses consisting of Arabian (*n* = 24), Fell pony (*n* = 21) and Akhal-Teke (*n* = 20). A variety of statistical population analysis techniques, such as principal component analysis (PCA), discriminant analysis of principal component (DAPC) and model-based method (STRUCTURE) were employed. The results of the population analysis clearly demonstrated a distinct separation of native and exotic horse breeds and clarified the relationships between studied breeds. The effective population size (Ne) for the last six generations was estimated 54, 49, 37, 35, 27 and 26 for the Caspian, Kurdish, Arabian, Turkmen, Akhal-Teke and Fell pony breeds, respectively. The Caspian breed showed the lowest LD with an average r^2^ value of 0.079, while the highest was observed in Fell pony (0.148). The highest and lowest average observed heterozygosity were found in the Kurdish breeds (0.346) and Fell pony (0.290) breeds, respectively. The lowest genomic inbreeding coefficient based on run of homozygosity (F_ROH_) and excess of homozygosity (F_HOM_) was in the Caspian and Kurdish breeds, respectively, while based on genomic relationship matrix) F_GRM_) and correlation between uniting gametes) F_UNI_) the lowest genomic inbreeding coefficient was found in the Kurdish breed. The estimation of genomic inbreeding rates in the six breeds revealed that F_ROH_ yielded lower estimates compared to the other three methods. Additionally, the Iranian breeds displayed lower levels of inbreeding compared to the exotic breeds. Overall, the findings of this study provide valuable insights for the development of effective breeding management strategies aimed at preserving these horse breeds.

## Introduction

The horse was domesticated between 5000 and 6000 years ago, marking a revolutionary shift in transportation and trade [1, 2]. Its rapid global dissemination was largely due to its remarkable versatility [3]. The Middle East, and Iran in particular, plays a crucial role in the study of early horse domestication. It is recognized as a vital geographic region where initial steps towards domestication are believed to have taken place. In these areas, social events, cultural diversity, and environmental conditions significantly influenced the development of various horse breeds, including the Caspian, Kurdish, Turkmen, Akhal-Teke, and Arabian horses [4]. Previous genetic studies aimed at deciphering the complexities of horse domestication have revealed that a considerable portion of the diversity found in modern maternal lineages, was already present at the time of domestication [1].

For effective management of equine resources, it is imperative to have a comprehensive understanding of breed characteristics. This encompasses data on population structure and effective size, linkage disequilibrium (LD), and inbreeding both within and across breeds. A holistic integration of these diverse data types will yield the most complete depiction of biological diversity within and among the breeds. Analysis of population structure and genetic diversity are vital for species evolution, conservation, and the success of breeding programs, underscoring its importance [4]. Nowadays, population genomic data from a wide range of organisms, make it possible to map basic evolutionary processes and evaluate genetic diversity via changes in effective population size (Ne) over the years [1]. In the context of livestock, a critical first step in studying genetic resources is to determine the effective size of the population and ascertain the rate of inbreeding [5]. The effective population size (Ne) is a genetic parameter that is instrumental in estimating inbreeding coefficients and is intricately linked to genetic diversity. This concept has been extensively researched across various species [6]. Inbreeding coefficients, initially dentified in humans, are based on various methods such as the Run of Homozygosity (ROH), Genomic Relationship Matrix (GRM), Excess of Homozygosity (HOM), and Uniting Gametes (UNI) [7]. Of these, ROHs are contiguous homozygous segments in the genome, characterized by the presence of identical haplotypes. Numerous studies have utilized genomic coverage for the precise identification of ROHs, contributing to the depth of our understanding in this field [8].

Iran is recognized as the reservoir of genetically unique breeds playing crucial roles in the livelihood of the human populations of this area. Three Iranian main horse breeds including Caspian, Turkmen and Kurdish were studied in the current paper. The Caspian horse is a small ancient breed, with its natural habitat is in the north of Iran, near the Caspian Sea. The Turkmen horse is thought to be the oldest surviving horse breed in the world, and is present largely on the north west of Iran. It was suggested that the Turkmen and Caspian horses might be ancestral to all forms of the oriental horse [4]. The Kurdish horse originates from the west of Iran, an area characterized by mountainous topography and a moderately cold climate.

To date, there has been little investigation of the genomic diversity of native Iranian horse breeds. This study aimed to assess the population structure, genetic diversity, LD patterns, and genomic inbreeding using four methods (F_GRM_, F_HOM_, F_UNI_, F_ROH_) across three main Iranian native breeds, as well as, three selected exotic horse breeds. The inclusion of various indigenous and certain exotic breeds for analysis is grounded in their shared characteristics and similarities, providing a robust foundation for this investigation (see Table 1). Considering their geographic proximity and annual migrations, it can be hypothesized that Iranian indigenous horse breeds such as Kurdish and Turkmen were closely associated with exotic breeds like Arabian and Akhal-Teke, respectively. In a similar vein, the grouping of Caspian and Fell Pony breeds in close proximity can be attributed to their comparable traits and performance characteristics. The outcomes of this study are anticipated to shed light on the similarities between native breeds, and the distinctions from their exotic counterparts. This newfound knowledge will be instrumental in formulating strategic plans for the breeding and conservation of equine resources.

**Table 1:** A summary of horse breeds used in the current study, their regions of origin, approximate population size, and their classification based upon use [1].

Breed	Geographic origin	Population size	Classification
Turkmen	Persia	Less than 2500	Riding horse, Endurance
Akhal-Teke	Turkmenistan	3500	Riding horse, Endurance
Caspian	Persia	Less than 3500	Pony, Riding and driving
Fell pony	England	6000	Pony, Light, draft
Kurdish	Persia	2700	Riding horse, Endurance, POLO match
Arabian	Middle East	1 Million	Riding horse, Endurance

## Materials and method

### Ethics statement

All methods and animal care and handling procedures were allowed and approved by the University of Kurdistan Animal Care and Use Committee (No. 2019/1112). All efforts were carried out in accordance with relevant regulations to minimize any discomfort during blood collection. The authors also complied with the ARRIVE (Animal Research: Reporting of In Vivo Experiments) guidelines.

### Animal resources and genotyping

A total of 156 horses were included in this study, comprising 91 horses belonging to 3 Iranian native breeds consisting Caspian (n = 38), Turkmen (n = 24), and Kurdish (n = 29), as well as 65 horses belonging to 3 exotic breeds including Arabian (n = 24), Fell pony (n = 21) and Akhal-Teke (n = 20) (Table 1). The Iranian native sample were genotyped using illumina SNP70K Beadchip, which contains 65167 SNP markers, while others were genotyped by the Equine Genetic Diversity Consortium (EGDC) using the Illumina SNP50 BeadChip, which includes 54,602 SNPs [1]. The datasets were merged using the SNPs that were shared between the chips and underwent quality control using PLINK v1.09 software [9]. The genomic data quality control involved several steps: firstly, SNP markers with unknown genomic locations or those located on the sex chromosomes were excluded. Secondly, animals exhibiting more than 5% missing genotypes were removed. Finally, SNPs with a minor allele frequency (MAF) below 0.05 and call rates below 95% were excluded from the analysis [10].

### Population structural analyses

To identify existing population structures, population stratification analysis was studied using three methods including Principal Component Analysis (PCA), Discriminant Analysis of Principal Component (DAPC) and STRUCTURE methods in the current study.

To determine how the animals were allocated to the groups using all markers that passed quality control, and to understand the genetic structure of the studied populations, the PCA was performed using prcomp function in the R version 3.6.2 software (https://www.rdocumentation.org/packages/stats/versions/3.6.2/topics/prcomp). The DAPC analysis was performed using adegenet 3.4.1 in the R version 3.6.2. The first step was to transform all genotypes into uncorrelated variables using PCA, and all principal components were retained for downstream analysis [11]. The selection of the optimal number of K-means clusters in the DAPC analysis was determined using the Bayesian Information Criterion (BIC), ensuring the accurate representation of distinct genetic clusters within the studied equine populations [11]. Management of animal genetic resources and conservation biology heavily relies on the identification of population stratification analysis and genetic admixture between individuals and populations [12]. Bayesian clustering using STRUCTURE 2.3.4 [13], allows for the visual assessment of the differentiation between population based on allele frequencies and permits the detection of admixture, indicating mixed ancestry within samples. The membership coefficients for the horse breeds were calculated, utilizing 5,000 iterations as burn-ins and 50,000 repetitions to define clusters at various K values, ranging from K = 2 to K = 10.

### Genetic diversity, linkage disequilibrium and estimation of the historical N_e_

To estimate basic genetic diversity, we calculated the observed (H_o_) and expected (H_e_) heterozygosity using Zhdanova & Pudovkin’s method [14] with PLINK v.1.9. The linkage disequilibrium (LD) measures were estimated between pairs of loci by calculating the squared correlation coefficient (r^2^) using Hill & Robertson’s method [15] using Haploview v4.2 software. We also calculated the Ne using the SNeP software [16]. Bin determination system was used for mean values of r^2^ in the distances of 10,000 to 20,000,000 bp [16]. The relationship between LD and Ne was demonstrated using the method by Corbin [17]. With the adjustment made for sample size and gametic phase uncertainty, the equation used was as follows:

$${\text{N}}_{\text{T}(\text{t})}={\left(4\text{f}\left({\text{c}}_{\text{t}}\right)\right)}^{-1}(\text{E}{\left[{{\text{r}}^{\text{2}}}_{\text{adj}}|\;{\text{c}}_{\text{t}}\right]}^{-1}-\alpha )$$ Eq 1

Here, N_T(t)_ represents the effective population size t generations ago. f (c_t_) is the mapping function for estimating the recombination rate (c_t_). ct, is defined for a specific physical distance between markers, α is the correction factor for mutation occurrence [18] and r^2^_adj_ signifies the LD value adjusted for sample size and the gametic phase using the method proposed by Weir & Hill [19]:

$${{\text{r}}^{\text{2}}}_{\text{adj}}={\text{r}}^{\text{2}}-\left(\beta \text{n}\right)-1$$ Eq 2

In this equation, the squared correlation coefficient (r^2^) values span 0.01 to 20 Mb distances, which are then utilized to derive N_e_ for numerous past generations. Here, n denotes the number of sampled individuals. If the gametic phase is unknown, *β* is set to 2; otherwise, it is set at 1.

The effective population size (N_e_) can also be calculated from r^2^ values for each autosomal chromosome, based on the following equations [17]:

$$E\left(r^{2}\right)\approx \frac{1}{\left(4cN_{\varepsilon }+1\right)}$$ Eq 3

$$N_{e}=\left(\frac{1}{E\left(r^{2}\right)}-1\right)\cdot (1/4c)$$ Eq 4

Where N_e_ is defined for the effective population size, r^2^ is the measure of LD among SNP alleles per autosomal chromosome, and *c* is the recombination rate in physical distance between markers, which is equal to 1/2*c*.

### Runs of homozygosity (ROH) detection and inbreeding coefficient based on ROH (F_ROH_)

ROH was identified for each individual using PLINK v1.9 [9]. A sliding-window of 50 SNPs with two missing SNPs and one heterozygous genotype across the genome was used to scan individual SNP genotypes and detect homozygous segments [20]. The inbreeding coefficient based on ROH (F_ROH_) was calculated as the ratio of the sum of L_ROH_ over the L_AUTO_ [21]:

$${\text{F}}_{\text{ROH}}=\frac{{\text{L}}_{\text{ROH}}}{{\text{L}}_{\text{AUTO}}}$$ Eq 5

Where L_ROH_ is the sum of ROH per animal and L_AUTO_ is the total length of the autosomal genome covered by SNP markers (approximately 2.235 Gbp of the horse genome).

### Estimation of inbreeding coefficients (F_GRM_, F_HOM_, F_UNI_)

Additionally, three alternative inbreeding coefficients (F_GRM_, F_HOM_, F_UNI_) were computed utilizing the GCTA software denoted as F1, F2 and F3 respectively [22]. F_GRM_ is calculated based on the variance of the additive genotypes [23], F_HOM_ is determined by the excess of homozygosity, and F_UNI_ is derived from the correlation between gametes [24].

## Results

### Quality control and PCA

The outcomes of the quality control procedures conducted on each horse breed are presented in Table 2. Out of 156 animals from six horse breeds, all animals passed the quality control. Following data cleaning, a total of 47944, 36544, 36445, 34618, 48024, and 39745 autosomal SNPs passed the filtering criteria in the Turkmen, Akhal-Teke, Caspian, Fell pony, Kurdish, and Arabian horse breeds, respectively (Table 2). Furthermore, 34,289 SNPs remained as common SNPs across all breeds under study.

**Table 2:** A summary of quality control steps in Iranian and exotic breeds.

	Turkmen	Akhal-Teke	Caspian	Fell pony	Kurdish	Arabian
Number of animals	24	20	38	21	29	24
Animals excluded due to genotyping call rate<95%	0	0	0	0	0	0
Remaining animals	24	20	38	21	29	24
Number of SNPs	65,167	54,602	65,167	54,602	65,167	54,602
Excluding unknown and the sexual chromosomes SNPs	3,421	1,998	3,421	1,998	3,421	1,998
SNP excluded for call rate <95%	4,504	7,362	15,654	16,118	4,005	5,532
Excluding SNPs with MAF<%5	9,298	8,698	9,647	1,868	9,717	7,327
SNPs remaining	47,944	36,544	36,445	34,618	48,055	39,745

**Fig 1:**
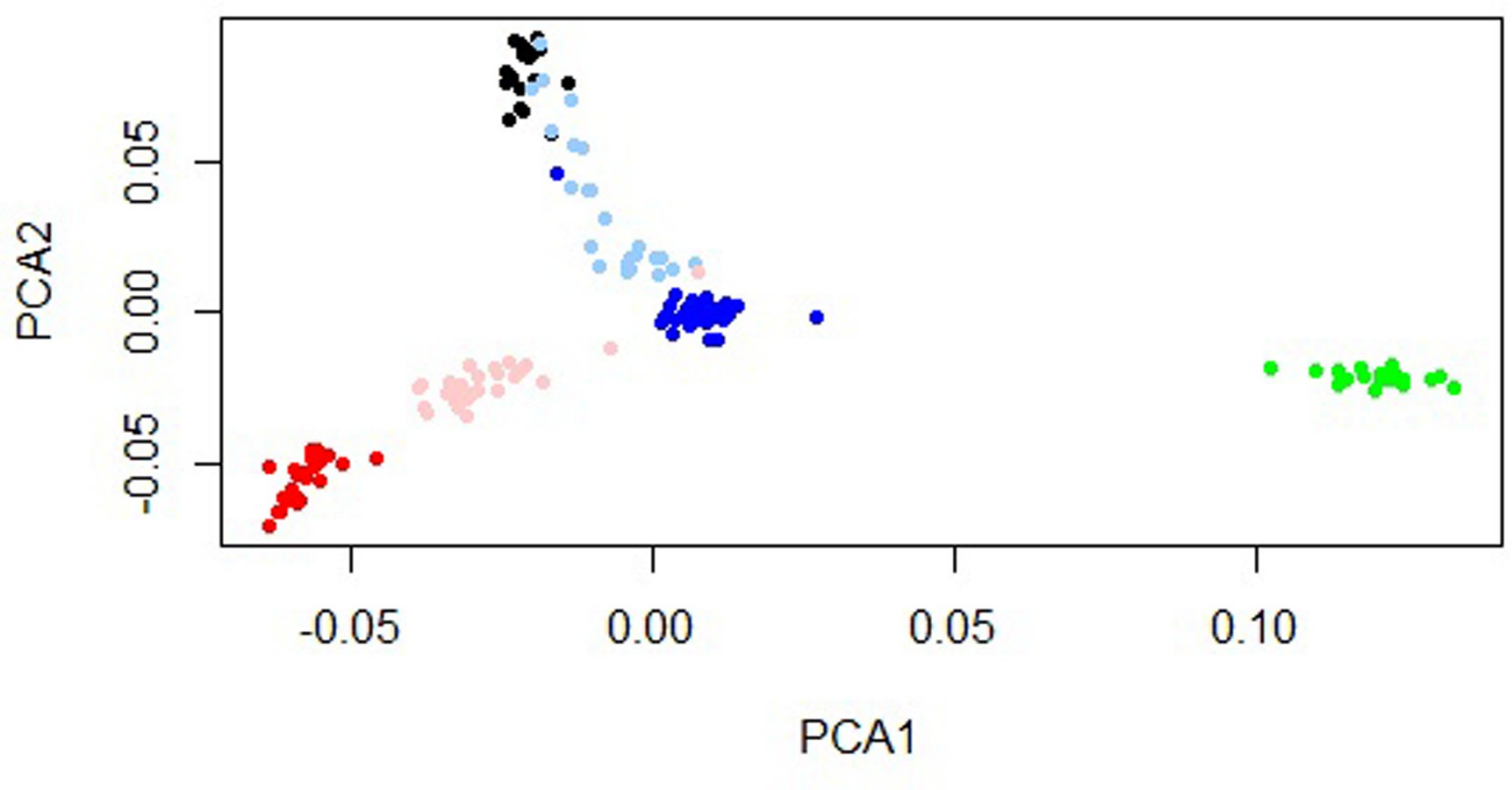
Animals clustered on the basis of principal components analysis (PCA). Colors are showing the individuals of black = Akhal-Teke, sky blue = Turkmen, blue = Caspian, green = Fell Pony, pink = Kurdish and red = Arabian breeds.

The scatterplot of the first two principal components is shown in Fig 1. The PCA results demonstrated that all the animals were accurately assigned to their respective original groups. The Fell Pony, Kurdish, and Arabian breeds were distinctly separated along PC1, whereas the Akhal-Teke, Turkmen, and Caspian breeds were differentiated along PC2. As illustrated in Fig 1, a small subset of animals including one from each of the Kurdish, Caspian, and Akhal-Teke breeds, alongside three from the Turkmen breed deviated from their true populations. These five animals were subsequently omitted from further analyses. In this analysis, the first and second components accounted for 8.48% and 5.23% of the total variation, respectively.

### Discriminant analysis of principal component (DAPC)

**Fig 2:**
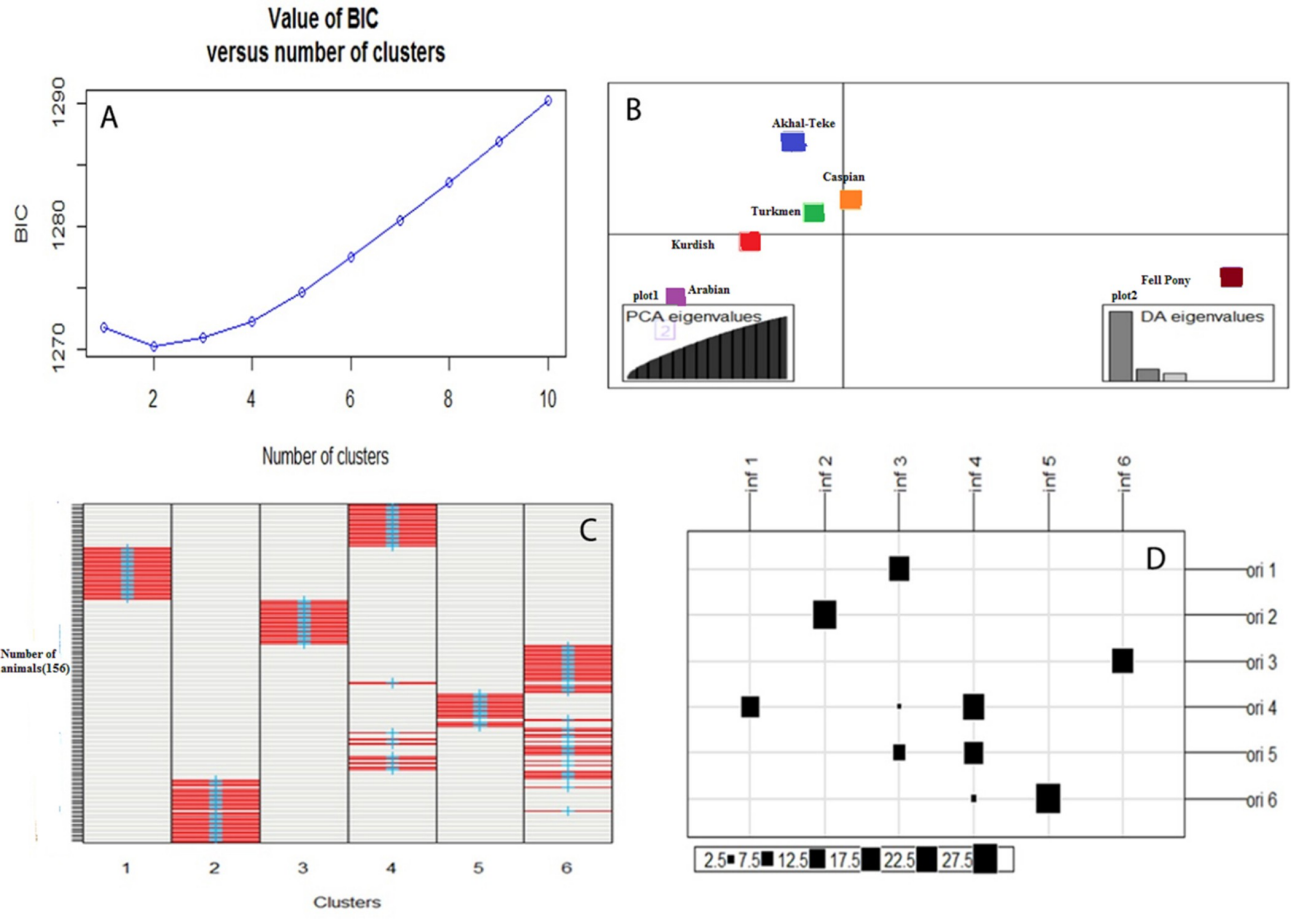
The results of the population structure analysis conducted using Discriminant Analysis of Principal Components (DAPC). Plot A showcases the relationship between Bayesian Information Criterion (BIC) values (displayed on the Y-axis) and the presumed number of K-means clusters (displayed on the X-axis). Plot B offers a visual representation of the DAPC clustering analysis for the six distinct breeds evaluated in this study. Within Plot B, Sub-plot 1 delineates the variance accounted for by the principal component (PC) axes utilized in the DAPC, while Sub-plot 2 elucidates the relative significance of the variance captured by the three discriminant axes. Plot C illustrates the accurate assignment of individuals (represented as rows) to their corresponding genetic populations, utilizing a color scheme to depict membership probabilities. Here, red symbolizes a probability of 1, white signifies a probability of 0, and blue crosses mark the initial cluster assignments determined by the K-means method. To provide clarity, the breeds of Akhal-Teke, Arabian, Fell Pony, Caspian, Turkmen, and Kurdish have been allocated to clusters 1 through 6, respectively. Plot D compares the actual groups (denoted as ’ori’) with the inferred groups (denoted as ’inf’), with the size of the black squares being directly proportional to the number of individuals allocated to various clusters.

Outcomes derived from the DAPC assessment are depicted in Fig 2. The evaluation of the optimal cluster number (K) ranged from 1 to 6, based on the Bayesian Information Criterion (BIC) statistic, is shown in Fig 2A. The analysis pinpointed K = 2 as the optimal cluster number, highlighted by the minimal BIC value attained.

Subsequent results from the DAPC animal clustering unveiled the formation of three distinct sub-populations (Fig 2B). A clear demarcation was noted, with Iranian breeds forming separate groups from the exotic breeds. Within this classification, breeds such as Caspian, Turkmen, Kurdish, and Akhal-Teke demonstrated a tendency to cluster closely together. In contrast, the Arabian and Fell Pony breeds were categorized distinctly apart.

Fig 2C illustrates the accurate assignment of individuals to their respective genetic populations. The plot underlines the effectiveness of the process, with a notable majority of individuals correctly placed within their genetic groups. A particular observation from this plot is the higher admixture proportions within the Iranian breeds namely the Caspian, Turkmen, and Kurdish breeds when contrasted with their exotic counterparts.

The distribution of individuals in actual (Ori) versus inferred (Inf) clusters is depicted in Fig 2D. The exotic breeds, Akhal-Teke, Arabian, and Fell Pony, were assigned to actual groups 1, 2, and 3 respectively, but were placed into the inferred groups 3, 2, and 6, respectively. On the contrary, the Iranian native breeds Caspian, Turkmen, and Kurdish, designated to actual groups 4, 5, and 6, demonstrated a diverse distribution across inferred groups 1, 3, 4, and 5. This distribution reflects a shared ancestry and a substantial degree of admixture. Their close geographical proximity and potential gene flow, especially among breeds such as Caspian, Turkmen, and Akhal-Teke, are mirrored in their tight clustering patterns (Fig 2B) and individual placements (Fig 2D).

## STRUCTURE

**Fig 3:**
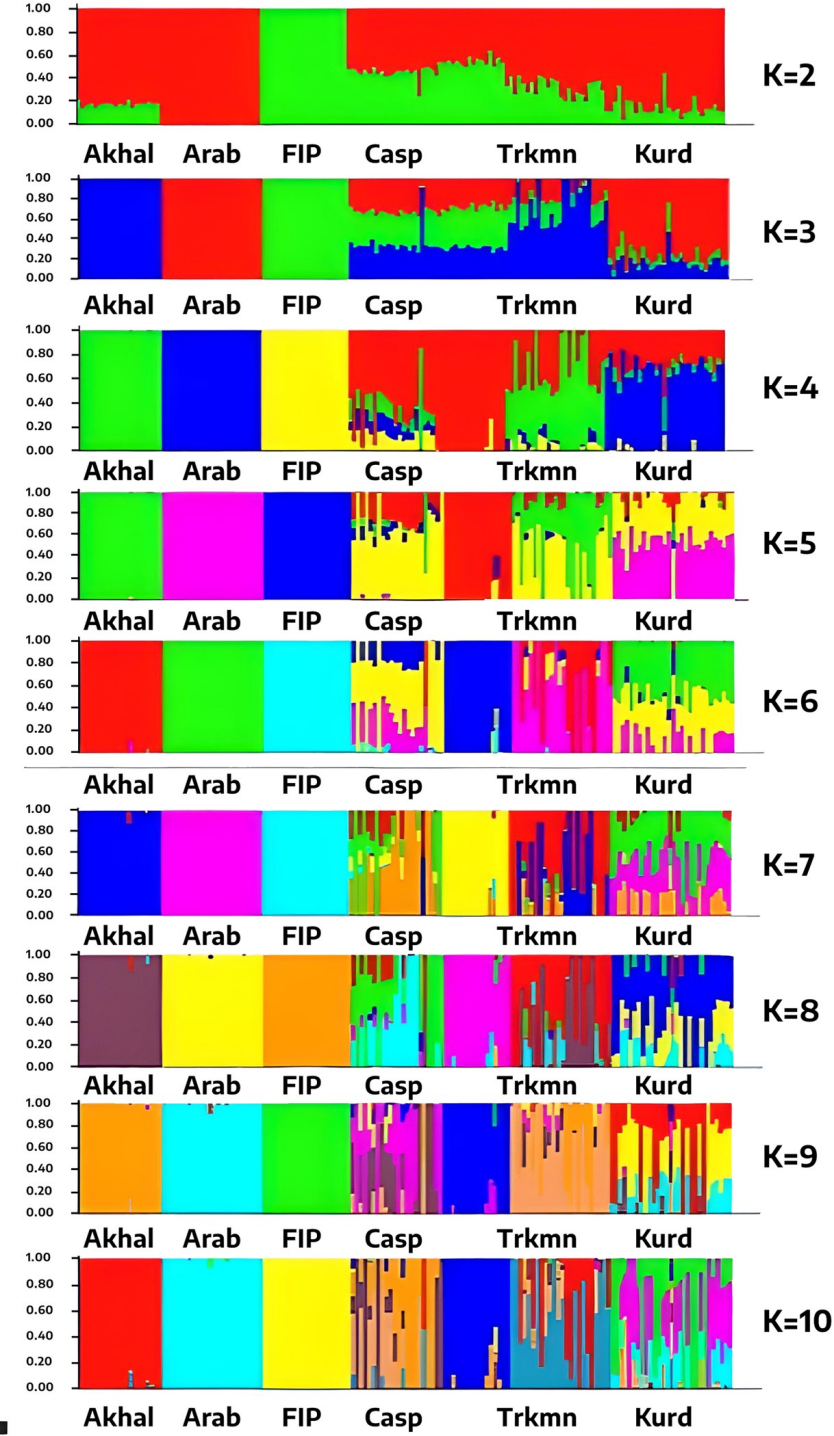
Cluster assignment for ten values of K values, as determined by STRUCTURE software. Each animal is represented by a vertical line, divided into K-colored segments, with each segment’s length reflecting the contribution from each inferential cluster. The breed abbreviations used in this figure are as follows: Akhal for Akhal-Teke, Arab for Arabian, FlP for Fell Pony, Casp for Caspian, Trkmn for Turkmen, and Kurd for Kurdish.

The clustering of individuals through STRUCTURE is depicted in Fig 3, with each individual represented as a vertical bar segmented into different colors, each denoting the individual’s estimated membership fractions. Individuals sharing the same color are categorized within the same cluster. STRUCTURE method is renowned for its capability to reveal the initial structural level within the dataset. Based on the rate of change of likelihood (ΔK), the optimal number of clusters was determined. This study identified two ancestral populations, with the peak value for the K criterion occurring at K = 2. This resulted in a noticeable level of admixture in the Iranian native samples, highlighting their genetic diversity. The six breeds under investigation were divided into two primary groups, with the Iranian and exotic populations collectively accounting for the total variance observed in this dataset. The Arabian and Fell Pony populations formed distinct clusters, whereas the Caspian, Akhal-Teke, Turkmen, and Kurdish breeds were grouped together, suggesting a shared ancestry or recent crossbreeding events among these horses.

At K = 3, the Iranian native breeds became more distinguishable, while the Fell Pony and Arabian horses predominantly aligned with the third cluster, setting them apart from the other horse breeds. As K increased to 5 or more, individuals from the exotic breeds consistently formed independent and distinct clusters. Specifically, at K = 6, K = 8, and K = 10, the Fell Pony breed consistently emerged as a unique group, corroborating the findings from our DAPC and PCA analyses.

### Genetic diversity

The results of expected and observed heterozygosity, two widely used measures of genetic diversity in various horse breeds are presented in Table 3. The average observed heterozygosity (H_o_) across breeds, calculated based on autosomal chromosomes, ranged from 0.2907 to 0.3458, while the average expected heterozygosity (H_e_) was 0.3457 to 0.3460. The findings from this study highlighted that Iranian horse breeds, including Kurdish, Turkmen, and Caspian, exhibited higher genetic diversity compared to the exotic breeds of Akhal-Teke, Arabian, and Fell Pony.

**Table 3:** Mean expected and observed heterozygosity, effective population size, and average r^2^ values in different horse breeds.

Characteristic	Akhal Teke	Arabian	Fell pony	Caspian	Turkmen	Kurdish
Expected Heterozygosity (H_e_)	0.3457	0.3460	0.3459	0.3457	0.3460	0.3458
Observed Heterozygosity (H_o_)	0.3257	0.3172	0.2907	0.3306	0.3375	0.3458
Ne (5 Generations ago)	27	37	26	54	35	49
Average of r^2^	0.1449	0.1441	0.1483	0.0791	0.1108	0.1006

#### Effective population size (N_e_)

**Fig 4:**
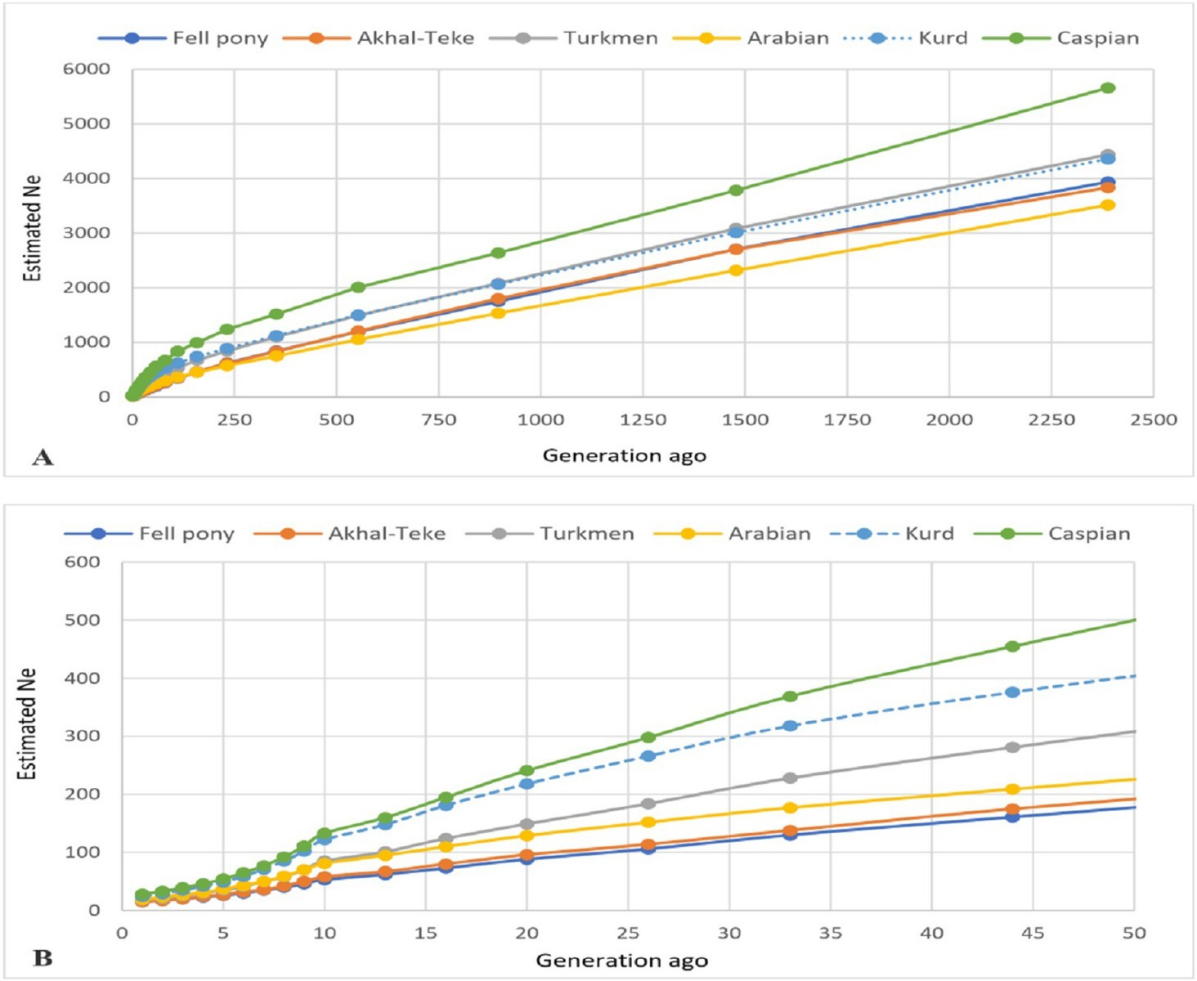
Effective population size (N_e_) trend plotted against generations in the past. Fig A: Distance range from 1 to 2500 generations ago. Fig B: Distance range from 1 up to 50 generations ago.

The results pertaining to the effective population size (N_e_) revealed a declining trend over the past 2,500 generations across all horse breeds, with a more rapid decrease observed approximately 50 generations ago. This trend was particularly pronounced for the Caspian breed, as depicted in Fig 4A.

In the last five generations, the N_e_ of Iranian native breeds was estimated to be around 54 for the Caspian, 49 for Kurdish, and 35 for Turkmen (Table 3 and Fig 4B). On the other hand, the exotic breeds exhibited lower Ne values, with 37 for Arabian, 27 for Akhal-Teke, and 26 for Fell Pony. Notably, our results revealed that the Turkmen breed (N_e_ = 37) and Fell Pony (N_e_ = 26) demonstrated the smallest effective population sizes among the Iranian and exotic horses, respectively (as shown in Table 3 and Fig 4B).

#### Linkage disequilibrium

**Fig 5:**
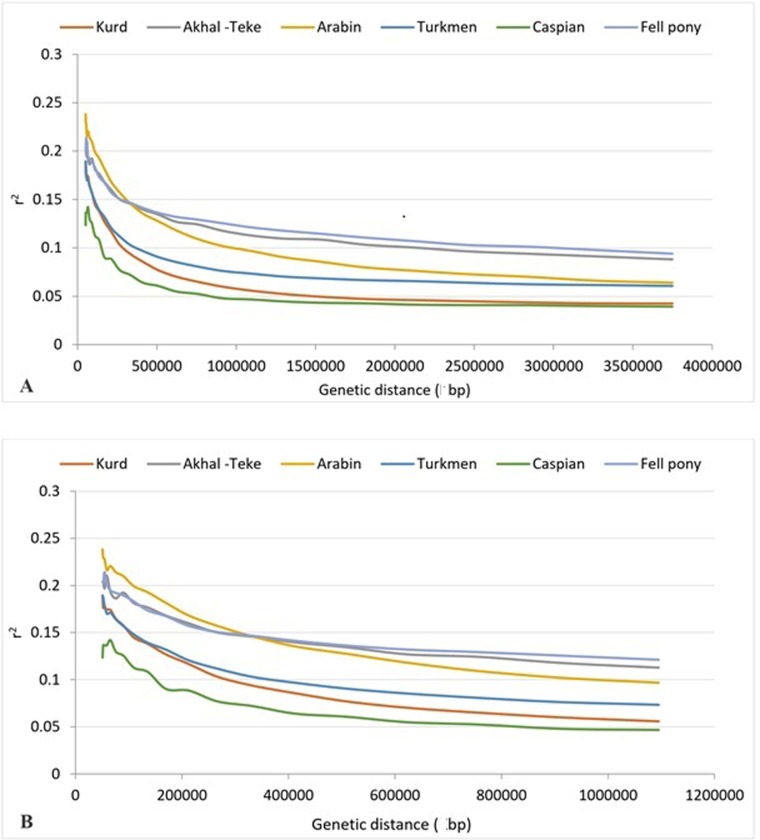
Fig A presents the LD decay map measured by r^2^ between SNP pairs according to distance with all chromosomes, distance range from 0 to 3750 Kbp and Fig B shows the LD decay map for the distance range from 0 to 1100 Kbp.

Linkage disequilibrium (LD) was computed for each breed individually, utilizing the average r² values. Fig 5 illustrates these averaged r^2^ values, showcasing a trend where LD tends to diminish as the distance between pairwise single nucleotide polymorphisms (SNPs) increases. A noticeable and rapid decline in LD was apparent within the initial 1Mbp. When comparing the Iranian native population to the exotic breeds, it was evident that the former displayed a significantly shorter distance at which r^2^ decayed to below 0.2. The LD plot typically exhibited a decrease in r^2^ values, transitioning from 0.23 (range: 0.20–0.25) to 0.2 (range: 0.20–0.25), 0.18 (range: 0.15–0.20) and 0.12 (range: 0.10–0.15) as the distances between markers increased from 200kbp, 400kbp, 600kbp, 800 kbp, 1Mbp, and finally 1.2 Mbp, respectively (as displayed in Fig 5). Among the breeds analyzed, the Caspian breed demonstrated the lowest LD, with an average r^2^ value of 0.0791 (detailed in Table 3). It is worth noting that the average r^2^ values for markers 50 kbp apart showed significant variation, a trend clearly visualized in Fig 5.

### Inbreeding coefficients

Table 4 presents the estimators of genomic-based inbreeding coefficient (F_ROH_, F_GRM_, F_HOM_, and F_UNI_) calculated for different Iranian and exotic horse breeds. The estimated inbreeding values based on F_HOM_ and F_UNI_ were higher in Fell pony (0.1179 and 0.1327, respectively) compared to the other horse breeds. This is consistent with our previous results obtained by observed heterozygosity and Ne in this breed, both of which were lower in Fell pony. Conversely, the inbreeding values obtained by F_HOM_ and F_GRM_ were the lowest in Kurdish horse breed (0.0204 and 0.0068, respectively). These results further confirm the genetic diversity and Ne results, which indicate higher diversity in the Kurdish breed compared to other horse breeds (detailed in Table 4).

**Table 4:** The mean of genomic inbreeding calculated by F_GRM،_ F_HOM_ F_UNI_ and F_ROH_ methods in different horse breed.

Breed	F_ROH_	F_UNI_	F_HOM_	F_GRM_
Akhal-Teke	0.0164	0.0739	0.0335	0.1143
Arabian	0.0507	0.0926	0.1135	0.0720
Fell pony	0.0237	0.1179	0.1327	0.1031
Caspian	0.0108	0.0366	0.0316	0.0415
Turkmen	0.1170	0.0248	0.0151	0.0344
Kurdish	0.0154	0.0204	0.0339	0.0068

## Discussion

In this study, three distinct methods including PCA, DAPC, and STRUCTURE were utilized to investigate the population structure. PCA is commonly employed in scenarios where the classes are not clearly defined, and animals from the same breed tend to cluster closely together on PCA plots [13]. In the PCA analysis conducted, it was observed that closely related breeds clustered together, with the Akhal-Teke and Turkmen breeds, serving as prime examples. On the other hand, Fell Pony horses were clearly differentiated from all other breeds. This observation aligns with the findings of previous studies, which have highlighted a distinct separation between oriental horse breeds and ponies [25].

While both PCA and DAPC rely on the extraction of principal components, their primary objectives differ. PCA focuses on capturing the maximum variance in the dataset, while DAPC aims to emphasize the separation between predefined groups, making it more suitable for classification tasks. The Population clustering of animals and breeds further evaluated using DAPC, which indicated that 2 clusters (K = 2) provided the optimal number of clusters in the dataset as evidenced by the lowest Bayesian Information Criterion (BIC) values. This suggests that all the studied breeds were categorized into two main groups, with the Iranian and exotic populations accounting for the total variance in the data. While few studies have reported the use of DAPC in different horse breeds, King et al. [26] ran DAPCs using 4–11 groups to analyse population structure in feral horses residing in certain areas of Little Book Cliffs Herd Management Area, Colorado USA. Their findings revealed that a scatter plot of DAPC results based on 5 clusters (BIC = 109.51) produced the most distinct groups when visually assessed. These 5 groups did not correspond to the 5 genetically distinct groups determined from residence in subjective areas, indicating that there is genetic exchange among areas within the population [26]. While our analysis identified two distinct clusters among the studied equine populations, the study conducted by King et al. [26] demonstrated the complexity of population structure in horses, emphasizing the importance of thorough genetic assessments in capturing the complex patterns of equine populations across diverse geographic regions.

The STRUCTURE results also supported the findings from the DAPC and PCA analyses. Iranian breeds were grouped together, suggesting a possible origin of these breeds from the same geographic region. The Caspian and Kurdish breeds exhibited the highest rate of admixture among the studied breeds. Individuals from the Kurdish, Arabian, Akhal-Teke, and Turkmen populations also demonstrated genomic similarities. Salek Ardestani et al. [27] reported that when K = 2, all of 37 horse breeds studied using whole genome sequencing data, were categorized into four main groups. These groups include the following: (1) Thoroughbreds and sport breeds; (2) Noriker, Saxon-Thuringian Heavy Warmblood, Percheron, Friesian, Sorraia, Dülmen pony, Connemara ponies, Welsh pony, and Jeju ponies; (3) American Miniature and Shetland ponies; and (4) Standardbreds, Arabians, and Akhal-Teke. Their results regarding Arabian and Akhal-Teke confirm our findings, showing similiraties between these exotic breeds. Overall, based on the PCA, and DAPC, and Structure analyses conducted in this study, it can be hypothesized that the Iranian indigenous horse breeds including Caspian, Kurdish and Turkmen (and Akhal-Teke) were closely grouped. Their proximity on the scatterplot suggests a shared ancestry, which is plausible considering the historical migrations of some nomadic communities to various regions [28].

Based on the outcomes related to genetic diversity, encompassing aspects such as expected and observed heterozygosity, linkage disequilibrium (LD) decay, and effective population size (N_e_), it was discerned that the Kurdish breed exhibited the highest genetic diversity (H_o_ = 0.34) amongst the Iranian breeds. This is likely attributable to lack of intense artificial selection, as the limited evidence of artificial selection within Iranian horse breeds suggests a comparatively lower degree of human intervention in their breeding, reflected in the higher genetic diversity. Conversely, the lowest level of genetic diversity estimates were for the Fell Pony and Arabian breeds in our study. Moradi and Khaltabadi Farahani [28] reported that H_e_ values within five Asian horse populations, encompassing Arabian, Akhal-Teke, Mongolian, and Caspian breeds were ranged from 0.27–0.30 [28]. Sadeghi et al. [29] delved into the genetic diversity in 71 Arabian horses and reported H_e_ and H_o_ values of 0.45 and 0.43, respectively. Furthermore, Almarzook et al. [30] employed genome-wide SNP data to investigate Syrian Arab horse breeds, discovering average H_e_ and H_o_ values ranging between 0.30–0.31 and 0.30–0.32, respectively. These findings suggest that a diminished level of heterozygosity in this horse breed might be a consequence of intense artificial selection targeting economically significant phenotypic traits or the isolation of populations that could have led to a reduced gene flow, leading to a loss of genetic diversity [28].

By definition, N_e_ is fundamentally a reflection of genetic diversity. The observed decrease in Ne is likely indicative of a reduction in genetic diversity and an increase in the rate of inbreeding, driven from differet factors like artificial breeding technologies, breed domestication, and formation. The results for N_e_ were aligned with the observed heterozygosity in both Caspian and Kurdish horse breeds, where a surge in genetic diversity is reflected by a higher N_e_. In contrast, the Fell Pony breed, which was identified as having the lowest genetic diversity, also displayed the smallest effective population size. Furthermore, an analysis of the historical trend of effective population size revealed a consistent linear decline across the studied breeds, mirroring the trends reported in the Thoroughbred horse breed [17].

The investigation of the LD is pivotal for genome-wide association studies as it facilitates the characterization of marker density required for the population under study. In the results obtained from this investigation, the Kurdish and Caspian breeds exhibited the highest persistence of the LD, followed by the Arabian, and then the Iranian breeds, particularly noticeable in the Turkmen breed. The results obtained for averaged r^2^ in LD depicted a decline with the increasing distance between pairwise SNPs, showcasing a rapid downtrend within the initial 1Mbp. Schaefer et al. [31] highlighted a pronounced decrease in LD within the first 1Mbp, with the most significant drop observed in the ponies and the slightest in the Thoroughbred breed. Salek Ardestani et al. [27] observed a heightened intensity of LD reduction within the first 0.5Mbp, which stabilized prior to reaching 1 Mbp and noted a more marked reduction in non-sport horses in comparison to sport horses. Lee et al. [32] also documented a sharp LD decrease in the first 1Mbp, which was followed by a more slower decrease untill to 3Mbp in Jeju horses. It should be noted that variations in the LD pattern between different studies may be due to different sample size and population structure, as well as differences in effective population size, share of haplotypes, selection processes, the markers utilized, and the average distances between SNPs, all of which could contribute to different LD [33].

Inbreeding coefficient estimates in horses are predominantly reported based on pedigree information. Gharahveysi et al. [34] reported that the average inbreeding coefficients to be 2.12% across the entire population of the Arabian horses in Iran. However, given the challenges of incomplete pedigree information and unreliable recording data available in most horse breeds, the application of genome-wide data and varied methodologies is preferred for more accurate estimation of inbreeding coefficients. A comparison between F_ROH_ and pedigree data in commercial pigs demonstrated that genomic data provides a more reliable basis than pedigree information [35].

The use of runs of homozygosity (ROH) leads to accurate estimation levels of autozygosity among individuals showcasing enhanced accuracy in distinguishing between identical by descent (IBD) and identical by state (IBS) segments. When compared to methodologies derived from GCTA software, ROH demonstrates less sensitivity to allele frequencies and can effectively distinguish between IBD and IBS segments [36]. F_ROH_ results indicate that the Iranian Caspian horse breed exhibits the lowest inbreeding values, boasting the largest effective population size (N_e_) compared to other horse breeds under study. In contrast, the Turkmen breed presents the highest genomic inbreeding values, a phenomenon attributable to intense artificial selection for competitions purposes, which is less prevalent in Caspian breeds. The average F_ROH_ values of 0.08, 0.11, and 0.042, has been previousy reported by Moradi and Khaltabadi Farahani [28] for Akhal-Teke, Arabian, and Caspian, respectively. These results were consistent with our findings, wherethe Caspian breed, exhibits the lowest estimated inbreeding, and the Arabian horses display higher inbreeding levels than other breeds.

Limited studies have reported genomic inbreeding coefficients across diverse horse breeds. Zanella et al. [35] suggested the sensitivity of averaged inbreeding estimations, as obtained through F_HOM,_ F_UNI_ and F_GRM_ to allele frequencies and the number of reference alleles copies for each SNP. Furthermore, these methods cannot distinguish between alleles that are IBD and IBS [35]. Polak et al. [37] reported average F_ROH,_ F_GRM_ and F_UNI_ values of 0.058, 0.004, and 0.064, respectively, in Sztumsi breed, alongside values of 0.0636, 0.0009, 0.0068, respectively, in Sokolski breed. Druml et al. [38] determined that the estimated mean of F_ROH_ in Polish Konic breed ranged between 0.15 to 0.27.

The Iranian breeds showcased lower estimated inbreeding (across four methods) compared to the exotic breeds. This discrepancy can be attribute to the small effective population size of the exotic breeds (notably the Fell pony) over the last five generations or to intensive artificial selection in exotic breeds comparing to Iranian horse breeds. In general, combination of increased inbreeding per generation and reduced effective population size can adversely affect genetic diversity within and between populations [28, 38, 39]. Consequently, breeding programs must vigilantly monitor the genetic variability to safeguard against irreversible erosion of genetic diversity.

Overall, this study represents a significant stride towards creating a valuable resource for genome-wide evaluation of structure and variations in indigenous and exotic horses. While the number of animals utilized in this study may be a limitation, it is vital to acknowledge that this dataset is the sole genomic information presently available for Iranian local horse breeds. Future research, encompassing a larger number of genotyped samples, is expected to unveil new insights into the domestication events and history of Iranian horses. Additionally, further investigation into phenotypic associations will enhance our understanding of the role that variations play in the development of local horse breeds.

## Conclusion

This study offers an exhaustive evaluation of population structure, alongside various genetic diversity parameters such as effective population size, linkage disequilibrium, expected and observed heterozygosity, and genomic inbreeding coefficients in select Iranian and exotic horse breeds. Analyzing the population structure through three distinct methods revealed the existence of two genetic clusters. The Bayesian clustering conducted by DAPC was consistent with the PCA and STRUCTURE. The estimation of effective population size indicated a decrease over the past generations, particularly in the Iranian Turkmen and exotic Fell pony, which exhibited the lowest effective population sizes among Iranian and exotic horse breeds. This emphasizes the necessitates of implementing breeding strategies, such as the identification and utilization of purebred horses, as well as designing an appropriate mating system. The analysis of inbreeding coefficients suggests that intensive selective strategies and the lack of mating systems with the aim of minimizing inbreeding depression are potentiall factors contributing to the deviation of populations from Hardy-Weinberg equilibrium, leading to reduced effective population sizes and genetic diversity and increased inbreeding across generations. These findings align with the F_ROH_ values observed in the Turkmen and Fell pony breeds in this study, highlighting the importance of implementing a proper breeding program to conserve these valuable native horse breeds.
